# The Growth and Scope of Voluntary Hospitals in London

**Published:** 1897-11-20

**Authors:** 


### The Institutional Workshop.

### THE GROWTH AND SCOPE OF VOLUNTARY HOSPITALS IN LONDON.

(From a Correspondent.)

### XV.—HOSPITAL DEVELOPMENT UNDER QUEEN VICTORIA.

The history of hospital progress during this reign is the history of medical, surgical, scientific, and social advance. Every new discovery, every achievement of the student, the mechanician, and the architect has been woven into the intricate organisation of London's voluntary hospitals. It is no easy task even at such a short distance of time to read aright the causes which have affected a growth so essentially popular as that represented in the rapid spread of such institutions during the last sixty years. It is no slight aid, however, in the elucidation of the hidden springs of benevolence to trace in chronological order the founding of hospitals, general and special, during this period. From the first rise of voluntary hospitals, which may be placed at 1719, the date of the founding of Westminster Hospital, to the commencement of this reign is a period of 118 years. Judged merely numerically with Victorian hospitals, the entire list of institutions founded during this 118 years appears insignificant. Twelve general and 12 special hospitals, together with 35 free dispensaries, make up the total of pre-Victorian institutions in London for medical relief. During the 60 years of this reign there have been added to this number 14 general hospitals, and no fewer than 47 special hospitals, besides 37 dispensaries. But it will be found that, with few exceptions, it is the older institutions which have attained to the greatest importance, whether of size or influence, and the actual accommodation provided for patients during these two periods must be compared before a just estimate can be obtained of hospital growth in either. In 1896, exclusive of the three endowed hospitals, the number of beds available in Metropolitan general hospitals was 4,002, and in special hospitals 3,578. Of these beds 1,193 alone belong to general hospitals, and 2,586 to special hospitals, founded within this reign. It is true that much of the expansion which has placed the older institutions in the front has taken place during this time. It will be convenient to study the growth of these institutions in decades.

1837—1846 Inclusive.

*General Hospitals.* (3.)

1839. King's College Hospital.
1845. St. Mary's.
1845. German Hospital.

*Special Hospitals.* (5.)

1838. Royal Orthopædic Hospital.
1841. Hospital for Consumption, Brompton.
1841. Hospital for Diseases of the Skin.
1842. Hospital for Women, Soho.
1843. Central London Ophthalmic Hospital.

1847—1856 Inclusive.

*General Hospitals.* (3.)

1849. London Homœopathic.
1856. Great Northern Central.
1856. West London.

*Special Hospitals.* (7.)

1847. Samaritan for Women and Children.
1848. City of London for Diseases of the Chest.
1850. City Orthopædic Hospital.
1851. Cancer Hospital, Brompton.
1852. Hospital for Sick Children, Great Ormond Street.
1854. Poplar Hospital for Accidents.
1856. Western Ophthalmic Hospital.

1857 to 1866 Inclusive.

*General Hospitals.*

None.

*Special Hospitals.* (15.)

1857. National for Diseases of Heart and Paralysis.
1857. Royal Eye Hospital.
1858. Dental Hospital of London.
1859. National for the Paralysed and Epileptic.
1860. North London for Consumption.
1860. St. Peter's for Stone, &c.
1861. National Dental Hospital.
1863. Hospital for Diseases of the Throat.
1863. St. John's for Diseases of the Skin.
1863. Lock Hospital (Male).
1864. British Hospital for Skin Diseases.
1864. Central London for Diseases of the Skin.
1866. Victoria Hospital for Children.
1866. Grosvenor for Women and Children.
1866. Hospital for Epilepsy and Paralysis.

1867—1876 Inclusive.

*General Hospitals.* (2.)

1867. French Hospital.
1873. London Temperance Hospital.

*Special Hospitals.* (8.)

1867. Alexandra for Children with Hip Disease.
1867. Belgrave for Children.
1867. North-Eastern for Children.
1868. East London Hospital for Children.
1869. Evelina Hospital for Sick Children.
1871. Chelsea Hospital for Women.
1872. New Hospital for Women.
1873. Central London Throat, Nose, and Ear.

1877 to 1886 Inclusive.

*General Hospitals.* (4.)

1878. North-West London.
1883. Miller Hospital.
1884. Italian Hospital.
1884. St. John's Hospital, Lewisham.

*Special Hospitals.* (6.)

1877. St. John's Lying-in House.
1878. West End for Diseases of Nervous System.
1878. Leyton and Walthamstow for Children.
1883. Paddington Green Children's Hospital.
1884. The Mothers' Lying-in Home.
1884. Gordon Hospital for Fistula.

1887 to 1896.

*General Hospitals.* (2.)

1890. West Ham, Stratford, and South Essex Hospital.
1892. Mildmay Mission Hospital.

*Special Hospitals.* (6.)

1887. London for Diseases of Throat, Nose, and Ear.
1887. Her Majesty's Hospital for Children and Adults.
1889. Clapham Maternity Hospital.
1889. Western Skin Hospital.
1890. British Hospital for Mental Diseases.
1894. Plaistow for Children.

It has not been possible to include all the minor institutions which, by the aid of a few beds and a handful of subscribers, have constituted themselves "hospitals." These are not often of long continuance, and, as we have shown in the early history of the voluntary system, they have always formed a kind of fringe to the more wisely conceived and durable institutions.

